# Aerosol release, distribution, and prevention during aerosol therapy: a simulated model for infection control

**DOI:** 10.1080/10717544.2021.2015482

**Published:** 2021-12-28

**Authors:** Marc Mac Giolla Eain, Ronan Cahill, Ronan MacLoughlin, Kevin Nolan

**Affiliations:** a Aerogen Ltd, IDA Business Park, Galway, Ireland; b School of Medicine, UCD Centre for Precision Surgery, University College Dublin, Dublin, Ireland; c School of Pharmacy and Biomolecular Sciences, Royal College of Surgeons, Dublin, Ireland; d School of Pharmacy and Pharmaceutical Sciences, Trinity College, Dublin, Ireland; e School of Mechanical and Materials Engineering, University College Dublin, Dublin, Ireland

**Keywords:** COVID-19, aerosol therapy, viral infections, vibrating mesh nebulizer, fugitive emissions, Schlieren imaging, aerosol visualization

## Abstract

Aerosol therapy is used to deliver medical therapeutics directly to the airways to treat respiratory conditions. A potential consequence of this form of treatment is the release of fugitive aerosols, both patient derived and medical, into the environment and the subsequent exposure of caregivers and bystanders to potential viral infections. This study examined the release of these fugitive aerosols during a standard aerosol therapy to a simulated adult patient. An aerosol holding chamber and mouthpiece were connected to a representative head model and breathing simulator. A combination of laser and Schlieren imaging was used to non-invasively visualize the release and dispersion of fugitive aerosol particles. Time-varying aerosol particle number concentrations and size distributions were measured with optical particle sizers at clinically relevant positions to the simulated patient. The influence of breathing pattern, normal and distressed, supplemental air flow, at 0.2 and 6 LPM, and the addition of a bacterial filter to the exhalation port of the mouthpiece were assessed. Images showed large quantities of fugitive aerosols emitted from the unfiltered mouthpiece. The images and particle counter data show that the addition of a bacterial filter limited the release of these fugitive aerosols, with the peak fugitive aerosol concentrations decreasing by 47.3–83.3%, depending on distance from the simulated patient. The addition of a bacterial filter to the mouthpiece significantly reduces the levels of fugitive aerosols emitted during a simulated aerosol therapy, *p*≤ .05, and would greatly aid in reducing healthcare worker and bystander exposure to potentially harmful fugitive aerosols.

## Introduction

Since the 2003 outbreak of the Severe Acute Respiratory Syndrome CoronaVirus (SARS-CoV) and subsequent COVID disease in China, there has been an increased focus on understanding the modes of transmission of viral respiratory infections. This focus has intensified since the emergence of the novel coronavirus SARS-CoV-2 from Wuhan, China in late 2019, and the subsequent global pandemic that has ensued.

It has been widely established that viral respiratory infections, such as human avian influenza A (H5N1) (Malik Peiris et al., 2009) and Middle East Respiratory Syndrome (MERS) (Zumla et al., 2015), are transmitted between people through respiratory droplets generated by an infected person coughing, sneezing, or speaking (WHO, 1730; Li et al., 2020; Ong et al., 2020). If inhaled, these virus carrying aerosols can lead to life threatening illnesses such as acute respiratory distress syndrome (ARDS) (Acosta & Singer, 2020; Badraoui et al., 2020). Aerosol therapy is the primary mode of treatment in such illnesses. The form of aerosol therapy used will vary depending on patient type and the severity of the illness. Whilst highly effective, the treatment type used has the potential to generate additional aerosols by causing the patient to cough or sneeze (Simonds et al., 2010; O’Neil et al., 2017). These forms of treatment are referred to as aerosol generating procedures (AGPs) and have been known to cause nosocomial infection among healthcare workers and others in clinical settings (Loeb et al., 2004; Tran et al., 2012; Hunter et al., 2016). Thus, in a clinical setting, there is considerable risk of virus transmission, such as SARS-CoV-2, to caregivers, bystanders, and fellow patients. As such, it is imperative that infection control measures are implemented to ensure safe working and treatment environments.

Use of personal protective equipment (PPE) by caregivers is seen as one means of protection against the potential inhalation of infectious fugitive aerosols. However, preventing the release of said emissions at source would be the most effective means of protection. Using aerodynamic particle sizers (APSs) (McGrath et al., 2019a; Mac Giolla Eain et al., 2021) quantified the release of fugitive medical aerosols during aerosol therapy. These works demonstrated that the addition of capture filters on the expiratory ports of face masks and mouthpieces and use of vibrating mesh nebulizers over jet nebulizers yielded mass concentration levels similar to ambient levels. However, these studies did not account for the particulate that would be generated by a patient. Due to the wide range in particle sizes generated, from 0.01 to 1000 µm in diameter (Jackson et al., 2020), it is unknown as to whether these capture filters would prevent the release of both fugitive medical and patient derived aerosols.

Flow visualization techniques such as Schlieren imaging, particle image velocimetry (PIV), and computational modeling are tools that can provide valuable insights into the behavior and dispersion distances of fugitive aerosols from the various aerosol therapy devices and patient interfaces. The use of such tools has shown that nebulizer air flow, lung function, and interface type all significantly impact upon the dispersion distance of fugitive emissions (Hui et al., 2007; Tang et al., 2009; Hui et al., 2015; Takazono et al., 2021). However, a limiting factor with these types of tools is the field of view, computational power, and lack of quantitative information on fugitive aerosol particle concentrations and size distributions. Instruments such as particle sizers provide this qualitative data on aerosol particle concentration, mass, and diameter; however, unless they are positioned in the correct location, the data captured may not be particularly relevant and potential fugitive aerosol hot spots missed. By using these measurement techniques in combination, the advantages of both mitigate the disadvantage of each individual technique. To date, to the best of the authors’ knowledge, this has not been attempted previously.

The hypothesis under investigation in this work was whether the addition of filtration was an effective means to prevent the release and spread of fugitive aerosols, both patient and medical, during a standard aerosol therapy with an aerosol holding chamber and mouthpiece. The work used both flow visualization and particle characterization techniques to document and measure this. The effects of breathing pattern, supplemental air flow and filtration on fugitive aerosol emissions and characteristics were also examined.

## Materials and methods

**Figure 1.**
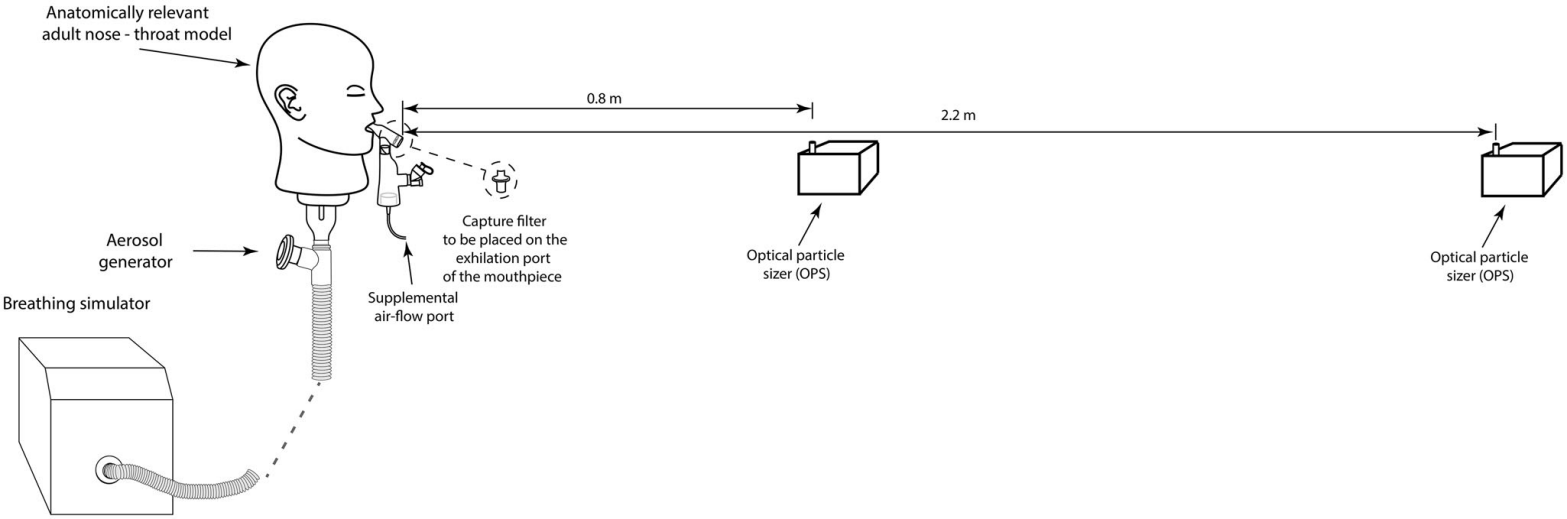
Schematic illustration of the experimental setup used to characterize the fugitive emissions.

A schematic illustration of the experimental setup used to measure the fugitive aerosol levels is presented in Figure 1. An anatomically relevant adult nose-throat model, previously described in Xi et al. (2014), Rygg & Longest (2016), and Bennett et al. (2018), was connected to an experimental breath actuated aerosol generator and a breathing simulator (BRS2100, Copley Scientific, Nottingham, UK). Simulated normal (tidal volume (Vt)=500 mL, breath rate (BR)=15 breaths per minute (BPM), and inspiratory:expiratory (I:E ratio)=1.0:1.0) (23) and distressed (Vt = 750 mL, BR = 30 BPM, and I:E = 1.0:1.0) (Réminiac et al., 2016; Dailey et al., 2017; Bennett et al., 2019) breathing patterns were used in this study. The novel experimental breath actuated aerosol generator mimicked the aerosol generated by a patient on the expiratory flow of the breath only. A saline solution (0.9% saline, BBraun, Dublin, Ireland) was aerosolized in the breath actuated aerosol generator to act as a tracer aerosol (Joyce et al., 2021; Sim et al., 2021).

A 2.5 mL dose of 1 mg/mL salbutamol (GlaxoSmithKline Ltd., Dungarvan, Ireland) was aerosolized using a vibrating mesh nebulizer (Aerogen Solo, Aerogen Ltd., Galway, Ireland) in combination with an aerosol holding chamber (Aerogen Ultra, Aerogen Ltd., Galway, Ireland). Salbutamol was chosen as it is a commonly nebulized formulation used in the characterization of aerosol delivery systems and is specified for use in ISO27427:^2013^. A standard mouthpiece was used to deliver aerosol from the aerosol holding chamber to the model. A removable bacterial filter (RespirGard II 303, Vyaire Medical Inc., Mettawa, IL) could be placed on the expiratory port of the mouthpiece. Supplemental gas flow rates of 0, 2 and 6 liters per minute (LPM), were considered. Supplemental air is often prescribed in combination with aerosol therapy to increase oxygen levels in the blood and ease the work of breathing of the patient (Fernández Fernández et al., 2021; Saeed et al., 2021). The flow rates considered in this study were those recommended as suitable for use by the manufacturer.

### Laser imaging

A 2D slice of the flow field was illuminated by a 2000 mW 532 nm continuous wave laser expanded through a cylindrical lens. The resulting light sheet was aligned with the sagittal plane of the model. A black sheet cloth provided a uniform dark background to help isolate light scattered by the aerosol from the background. The camera was placed orthogonal to the laser sheet so that the illuminated flow field could be quantified using the PIV method.

Video was recorded using a Canon EOS R5 camera at a resolution of 4096 × 2160 pixels (DCI 4K) at 29.97 frames per second (FPS). A Canon RF 85 mm f/1.2 lens was used; at an aperture of f/1.2 this lens is not diffraction limited and can resolve particles >4 µm at 4K resolution. This also ensures a uniform out of focus background so that the aerosol can be isolated in the image. PIV allows the quantification of the 2D flow field within the light sheet. The open source PIVLAB library for MATLAB (Thielicke & Stamhuis, 2014), was used to process the videos and obtain velocity fields.

### Schlieren imaging

Schlieren imaging exploits the local refraction of light by an inhomogeneous medium, such as ambient air. A Z-configuration (Settles, 2001) was employed comprised of two 400 mm diameter parabolic mirrors of focal length 1.8 m. A broadband white light LED (Thorlabs, Newton, NJ) was used for illumination and the light was focused on to a razor blade. A Canon 5D mk III camera with a EF-100 mm f/2.8 lens was placed downstream of the razor blade and used to record video at 1280 × 720 pixel resolution at 59.94 FPS. Flow features were extracted via sequential frame differencing to enhance the contrast of moving flow, and the Farneback optical flow technique was employed to estimate flow structure advection using MATLAB.

### Fugitive emissions characterization

Optical particle sizers (OPSs, model 3330, TSI Inc., Shoreview, MN) were used to measure aerosol particle number concentrations (PNCs) and particle size distributions, between 0.3 µm and 10 µm. Similar to other studies of this type (McGrath et al., 2019a, 2019b), the OPSs were positioned 0.8 m and 2.20 m from the end of the mouthpiece. Aerosol PNC and size distributions were measured at five second intervals for a total of 30 minutes. The 30-minute test consisted of an initial five-minute period to establish ambient conditions in the test room. The remaining 25 minutes consisted of dose nebulization, approximately seven minutes, and aerosol decay post nebulization. After each test, the room was ventilated and monitored to ensure ambient levels had been reestablished.

The use of the breath actuated aerosol generator with the aerosol treatment allowed for a more accurate simulation of the fugitive aerosols generated in a clinically relevant situation. Hence, the data reported represents a more accurate reflection of the fugitive aerosols levels that caregivers, other patients, and bystanders are exposed to. The methods used to measure and characterize the fugitive aerosols in this piece of work could not differentiate between the different types and sources of aerosol, i.e. fugitive medical or patient derived. As such, all the data that are presented and discussed in this piece of work are a combination of both fugitive medical and simulated patient derived aerosols and will be referred to under the blanket term of fugitive aerosol.

### Experimental test room

The laboratory room in which this study was conducted had dimensions of *L* = 8.2 m, *W* = 6.3 m, and *H* = 2.4 m, 124 m^3^. The mechanical ventilation in the test room was powered off for all testing. The test room had a single occupant who was positioned behind the breathing simulator so as not to affect the distribution of the fugitive aerosols. The air change rate was determined using the tracer gas decay method with CO_2_ as the tracer (Sherman, 1992). An indoor air quality probe (Direct Sense II multi-sensor probe, GrayWolf Sensing Solutions, Shelton, CT) was used to measure the CO_2_ levels. The air exchange rate was calculated to be approximately 1.15/h.

### Statistical methods

Results are expressed as the mean ± standard deviation of the particle numbers. Paired Student’s *t*-tests were conducted using the software package Minitab 19.20201.0 (Minitab LLC, State College, PA) to establish if the particle numbers released varied significantly with the different mouthpiece filtration and breathing patterns examined. *p* Values ≤.05 were considered statistically significant. The experiments were repeated three times independently (*n* = 3) for each test scenario.

## Results

### Flow visualization

**Figure 2.**
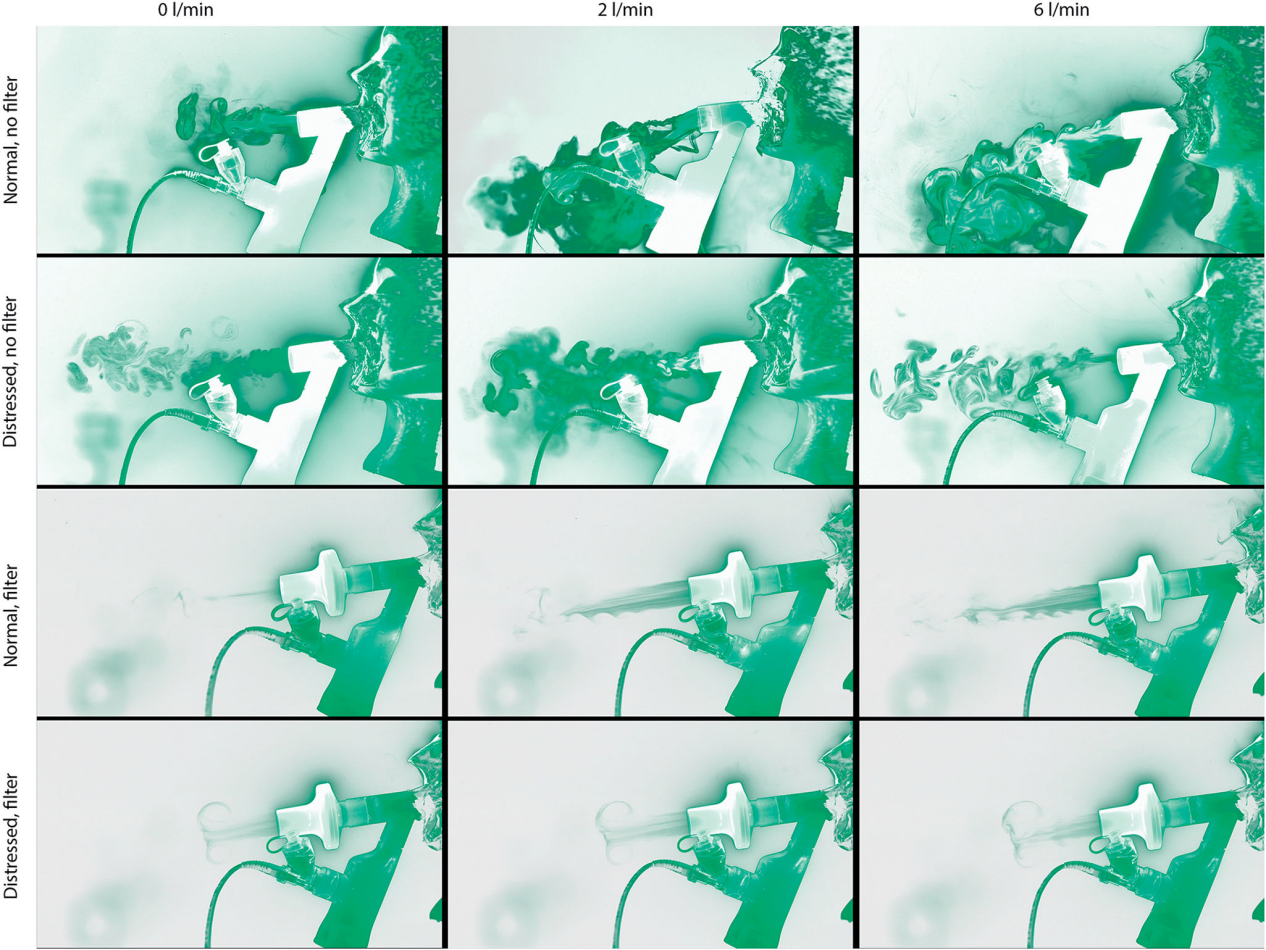
Collage of flow visualization results for the laser sheet images. Row and column headers indicated the flow rate, breathing type, and filter use. Note that the images have been partially inverted with a custom look up table which brightens the black background. This has the effect of increasing the viewing contrast of the aerosol.

Figure 2 shows a collage of representative image frames from the laser imaging video recordings. The color data have been modified with a look-up-table to invert the image intensity in the dark regions of the image while maintaining a linear response in the rest of the image. This has the advantage of greatly improving the visualization of the laser illuminated aerosol particles which would otherwise be difficult to discern against the black background. Rows 1 and 2 in Figure 2 show the release of the fugitive aerosols from the unfiltered mouthpiece and rows 3 and 4 show the release of fugitive aerosols from the filtered mouthpiece. The quantity of fugitive aerosol from the unfiltered mouthpiece is significant and increases as the supplemental air flow rate increases. The aerosol was observed to quickly fall toward the floor and out of the field of view of the camera. The addition of the filter to the mouthpiece, rows 3 and 4, greatly reduced the quantity of fugitive aerosol released into the test room. A distressed breathing pattern, characteristic of patients with respiratory difficulties, resulted in a lower quantity of fugitive aerosols released, rows 2 and 4, compared to a normal, quiet breathing pattern.

**Figure 3.**
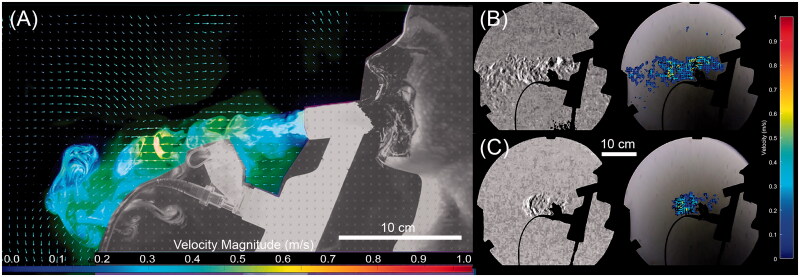
PIV and Schlieren visualizations of the flow structure for the unfiltered mouthpiece at six LPM. (A) PIV vector field for a normal breathing pattern. (B) Schlieren image of the flow at the same conditions showing contrast enhanced image and resulting optical flow velocity estimation and (C) Schlieren image of distressed flow at the same flow rate.

The results of the Schlieren visualization are presented in Figure 3 and are compared to the PIV analysis of the laser visualization. As detailed in the ‘Materials and methods’ section, Schlieren visualization requires a change in the refractive index, typically in the form of local temperature or species variation. Schlieren is not capable of imaging aerosol, conversely laser imaging only detects aerosol. As a result, this allows the decoupling of the air flow and aerosol trajectories. In the case of the filtered mouthpiece tests, there was no such change due to the homogenization of the field by the filter. Similarly, due to the effectiveness of the filter, there is insufficient particle to perform PIV analysis on the data. As such, the images presented in Figure 3 are for the unfiltered mouthpiece only. It is apparent that the aerosol initially follows a very close path to the expiratory port of the mouthpiece. However, this fugitive aerosol plume quickly falls toward the ground, within 0.2 m of the exit of the mouthpiece.

### Fugitive aerosol emissions

Table 1 presents the time-average PNC for the entire 30-min test duration for each test scenario. The average particle concentration in the test room prior to nebulization, i.e. ambient conditions, was 110.59 ± 13.75 (91.01–128.25) #/cm^3^. The data in Table 1 confirm the presence of fugitive aerosols in the test room during and post nebulization. The *p* values in Table 1 indicate that the addition of the filter to the mouthpiece made a statistically significant difference to the release and spread of fugitive aerosols, irrespective of breathing pattern and supplemental air flow rate. While there is an increase in the particle numbers above ambient, the recorded values are much closer in approximation to ambient than the unfiltered case.

**Table 1.** Time averaged particle number concentration for the 30-minute test duration.

		Average particle number concentration (#/cm^3^)					
		0.8 m			2.2 m		
Breathing pattern	Flow rate (LPM)	Filtered MP	Unfiltered MP	*p* Value	Filtered MP	Unfiltered MP	*p* Value
Normal	0	115.17 ± 0.77	154.82 ± 6.28	.009	115.03 ± 0.66	129.15 ± 2.51	.014
	2	129.25 ± 3.91	182.61 ± 7.70	.014	112.70 ± 0.70	148.86 ± 8.23	.027
	6	146.13 ± 5.13	187.85 ± 3.94	.001	126.99 ± 3.73	154.72 ± 8.09	.043
Distressed	0	115.80 ± 1.39	143.90 ± 4.83	.005	114.58 ± 0.51	129.66 ± 3.40	.021
	2	121.17 ± 1.41	144.51 ± 5.32	.025	122.45 ± 1.01	140.27 ± 3.65	.012
	6	114.83 ± 0.82	147.32 ± 5.95	.010	115.38 ± 0.36	140.38 ± 3.75	.018

The data in the table show the effects of mouthpiece filtration, breathing pattern, and distance have on fugitive aerosol levels. *p* Values are included in the table to highlight whether mouthpiece filtration had a statistically significant effect on reducing the levels of fugitive aerosols in the room, with *p* values ≤.05 considered significant.

Table 2 compares the average peak particle numbers measured for each of the different test scenarios. The data presented in Table 2 are the peak particle numbers above ambient levels (peak value – ambient value). Included in the table are the results of Student’s *t*-tests performed to determine whether there was a statistically significant difference in peak particle numbers in the test room with and without a filter on the mouthpiece. Irrespective of distance from the source, supplemental air flow rate through the therapy device and breathing pattern, the addition of a filter to the mouthpiece resulted in a statistically significantly lower level in fugitive aerosols in the test room.

**Table 2.** Average peak (±standard deviation) particle number concentration above ambient room levels.

		Peak particle number concentration (#/cm^3^) above ambient levels					
		0.8 m			2.2 m		
Breathing pattern	Flow rate (LPM)	Filtered MP	Unfiltered MP	*p* Value	Filtered MP	Unfiltered MP	*p* Value
Normal	0	25.00 ± 3.22	118.85 ± 12.87	.010	16.06 ± 2.21	67.96 ± 2.34	.000
	2	42.09 ± 3.71	119.33 ± 11.77	.004	54.79 ± 4.11	77.71 ± 3.76	.035
	6	81.27 ± 7.75	154.28 ± 9.15	.000	57.59 ± 2.18	111.12 ± 13.18	.017
Distressed	0	31.72 ± 2.76	82.03 ± 12.71	.027	15.56 ± 2.97	77.50 ± 7.12	.007
	2	38.42 ± 3.91	86.49 ± 4.32	.000	31.67 ± 4.40	64.91 ± 4.12	.001
	6	41.12 ± 7.00	113.01 ± 11.04	.002	13.00 ± 2.36	78.85 ± 4.44	.001

The data show the effects of mouthpiece filtration, breathing pattern, and distance on fugitive aerosol levels. *p* Values are included in the table to highlight whether mouthpiece filtration had a statistically significant effect on reducing the levels of fugitive aerosols in the room, with *p* values ≤.05 considered significant.

### Aerosol droplet sizing

Table 3 summarizes the average (±standard deviation) particle numbers below a threshold of 5 µm in diameter. This threshold was chosen as it has been documented in the literature that aerosol particles <5 µm in diameter carry the highest risk of airborne transmission of viral loads and inhalation risk (Agarwal et al., 2020; Liu et al., 2020; van Doremalen et al., 2020). The data in Table 3 highlight the effectiveness of the filter placed on the expiratory port of the mouthpiece in capturing particles below this threshold, with *p*<.05 indicating statistical significance.

**Table 3.** Average (±standard deviation) particle numbers below 5 µm particle diameter threshold for both filtered and unfiltered mouthpieces.

		PN ≤5 µm (#)			PN ≤5 µm (#)		
		0.8 m			2.2 m		
Breathing pattern	Flow rate (LPM)	Filtered MP	Unfiltered MP	*p* Value	Filtered MP	Unfiltered MP	*p* Value
Normal	0	109.67 ± 11.7	150.00 ± 7.02	.044	93.00 ± 7.00	123.33 ± 10.21	.036
	2	137.67 ± 8.08	181.67 ± 6.39	.042	129.33 ± 12.50	163.67 ± 12.01	.047
	6	272.67 ± 11.15	347.33 ± 11.37	.033	191.67 ± 5.86	266.33 ± 7.09	.005
Distressed	0	128.33 ± 8.50	156.00 ± 7.00	.045	95.33 ± 6.03	121.24 ± 10.00	.032
	2	125.12 ± 12.53	183.33 ± 8.33	.002	101.48 ± 6.08	171.33 ± 6.67	.007
	6	131.00 ± 2.65	334.67 ± 13.20	.002	122.00 ± 3.61	205.00 ± 8.19	.006

The data in the table highlight the effects of mouthpiece filtration, distance, and breathing have on fugitive aerosol levels in the room. *p* Values are included in the table to highlight whether mouthpiece filtration had a statistically significant effect on reducing the levels of respirable fugitive aerosols in the room, with *p* values ≤.05 considered significant.

## Discussion

This study examined the release of fugitive aerosols into the atmosphere during a standard medical aerosol treatment to a spontaneously breathing simulated adult patient. The study provided real-time qualitative and quantitative insights into the dispersion of the fugitive aerosols emitted from expiratory port of a standard mouthpiece used in aerosol therapy. The data from the current study show that during aerosol therapy the release of fugitive aerosols varies with breathing pattern, supplemental air flow rate, distance from the source and filtration. A distressed breathing pattern generates lower levels of fugitive aerosols compared to a normal breathing pattern. As the supplemental air flow rate to the aerosol holding chamber increases, the levels of fugitive aerosols generated increases. The potential exposure to fugitive aerosols decreases as distance from the source increases. The addition of filters to the exhalation port of the mouthpiece significantly reduces the levels of respirable fugitive aerosols released into the environment, *p*≤.05.

There are several factors that influence the quantity of fugitive aerosol released, including, but not limited to: device, patient type, and interface. While the concentration and dispersion is affected by the room layout, ventilation, size, temperature, and air turbulence (Long et al., 2001; Ciuzas et al., 2015). As such, it is necessary to consider these factors when analyzing the results of this study.

The addition of the capture filter to the expiratory port of the mouthpiece greatly reduced the release of fugitive aerosols into the atmosphere, with reductions in the peak values ranging from 47.3 to 77.7% for a normal breathing pattern and 61.3 to 83.6% for a distressed breathing pattern. Although the reductions were not as significant as those reported in other studies (Wittgen et al., 2006; Ari et al., 2016; McGrath et al., 2019a; Mac Giolla Eain et al., 2021), the images and data highlight the effectiveness of filters in limiting the release and spread of fugitive aerosols.

It can be seen from the flow visualization (Figure 2) that the greatest release of fugitive aerosols was from the unfiltered mouthpiece during simulated normal breathing. The greatest concentration of these fugitive aerosols was within 0.4 m of the end of the mouthpiece and is in agreement with other flow visualization studies (Hui et al., 2007, 2009, 2015). Moving beyond 0.4 m, the concentration of these fugitive aerosols decreases due to the highly chaotic, turbulent flow structure of the fugitive aerosol plume. As a result, the aerosol begins to move more laterally rather than centrally along the midline of the simulated patient. This observation from the flow visualization data correlates with the data from the particle sizers (Table 1), where the greatest number of particles were detected closer to the expiratory port, 187.85 ± 3.94 #/cm^3^ at 0.8 m and 154.72 ± 8.02 #/cm^3^ at 2.20 m. This is particularly relevant to healthcare workers as they are within this 0.4–0.8 m range, approximately arm’s length, when treating patients.

The addition of the filter to the mouthpiece greatly reduces the quantity of aerosol released (Figure 2, rows 3 and 4). The exhaled aerosol plume is much smaller and more ordered, similar to a jet in structure. This results in much less lateral spread of the aerosol. However, this much more coherent flow poses a greater risk of long-range aerosol transport longitudinally. This finding corroborates the particle number data presented in Tables 1 and 2, particularly at 0 and 2 LPM of supplemental air flow. These findings indicate that although the addition of the filter greatly reduces the quantity of fugitive aerosols, there is a risk that these droplets will deposit on surfaces beyond the 2.20 m range considered in this study and pose a risk of transmission through physical contact (Cortegiani et al., 2018). As such, appropriate precautions should be taken by healthcare workers, other patients, and bystanders.

The breathing pattern of the simulated patient also effected the release and dispersion of fugitive aerosols. The simulated distressed breathing patient generated lower numbers of fugitive aerosols than the normal, healthy breathing pattern (Tables 1 and 2). This is also clear from the images presented in the flow visualization part of this work (Figure 2). Although this result seems counterintuitive, works examining the effects of breathing pattern on aerosol drug delivery have found that delivery efficiency was greater in simulated distressed breathing compared to normal breathing (Bhashyam et al., 2008; Réminiac et al., 2016; Bennett et al., 2019). The studies attributed the greater aerosol delivery to the increased tidal volume and BR of the distressed breathing pattern. Consequently, healthcare workers should be aware that a patient who is not undergoing any breathing difficulties but has a potentially infectious viral infection would be a greater spreader of infection than others.

The addition of a capture filter on the expiratory port had a statistically significant effect on reducing the number of fugitive aerosol particles below the 5 µm critical threshold, *p*<.05. Furthermore, as the additional air flow rate increased the effectiveness of the filter was more apparent. The filters used in this study are rated at 99.9% bacterial and 99.8% viral efficiency of 0.3 µm or larger sized particles. Hence, the effectiveness of the filter in capturing particles <5 µm is unsurprising and should be, where possible, incorporated on the expiratory port of all respiratory therapy devices.

These data presented in this study highlighted a number of means by which healthcare workers, other patients, and bystanders could be exposed to potentially infectious fugitive aerosols in a clinical setting. The World Health Organisation (WHO) guidelines for infection prevention and control of acute respiratory infections in healthcare settings recommends that healthcare workers should wear a surgical mask, eye protection, and take contact precautions if within 2.0 m from a potentially infectious patient (WHO, 2014). However, this work suggests that healthcare workers should wear full airborne protective equipment, N95 mask or equivalent, gown, gloves, goggles, hair covers, and face shield or hood, in any enclosed setting, irrespective of distance, ventilation rate, and device filtration. Environmental cleaning to reduce contact transmission in health care facilities is also necessary.

There are a number of limitations to this study, the *in vitro* nature constitutes one of its main limitations. The room airflow was switched off during the experiments in order to reveal the maximum distribution of the fugitive aerosol without interference by external airflow. As such, the test room had a low air exchange rate. Further work is needed to assess the interaction between, for example, ward and ICU ventilation, on the dispersion of fugitive aerosols. A single aerosol-patient interface and therapy device was used in this study. Further research examining the different types of aerosol therapy-interface combinations is warranted.

## Conclusions

This study presents both qualitative and quantitative data that confirmed the release and dispersion of fugitive aerosols into the environment during a standard nebulizer treatment with an aerosol holding chamber and mouthpiece to a spontaneously breathing simulated adult patient. Our study shows that the addition of a capture filter to the expiratory port of the mouthpiece significantly reduces the release fugitive aerosols. A normal, relaxed breathing pattern leads to a greater release and spread of fugitive aerosols than a distressed breathing pattern. The additional of supplemental air flow to the aerosol holding chamber increased the levels of fugitive aerosols in the test environment. The addition of filters to the exhalation port of the mouthpiece significantly reduces the levels of respirable aerosols, ≤5 µm in diameter, *p*≤.05, and reduces the risk of exposure to healthcare workers and bystanders. The findings presented in this study could be used by healthcare organizations to inform policy and best practices for risk mitigation from fugitive aerosols.
